# Tail Risk Dynamics under Price-Limited Constraint: A Censored Autoregressive Conditional Fréchet Model

**DOI:** 10.3390/e26070555

**Published:** 2024-06-28

**Authors:** Tao Xu, Lei Shu, Yu Chen

**Affiliations:** Department of Statistics and Finance, School of Management, University of Science and Technology of China, Hefei 230026, China; xu0130@mail.ustc.edu.cn

**Keywords:** autoregressive model, censored data, entropic value at risk, extreme value theory, financial risk management

## Abstract

This paper proposes a novel censored autoregressive conditional Fréchet (CAcF) model with a flexible evolution scheme for the time-varying parameters, which allows deciphering tail risk dynamics constrained by price limits from the viewpoints of different risk preferences. The proposed model can well accommodate many important empirical characteristics of financial data, such as heavy-tailedness, volatility clustering, extreme event clustering, and price limits. We then investigate tail risk dynamics via the CAcF model in the price-limited stock markets, taking entropic value at risk (EVaR) as a risk measurement. Our findings suggest that tail risk will be seriously underestimated in price-limited stock markets when the censored property of limit prices is ignored. Additionally, the evidence from the Chinese Taiwan stock market shows that widening price limits would lead to a decrease in the incidence of extreme events (hitting limit-down) but a significant increase in tail risk. Moreover, we find that investors with different risk preferences may make opposing decisions about an extreme event. In summary, the empirical results reveal the effectiveness of our model in interpreting and predicting time-varying tail behaviors in price-limited stock markets, providing a new tool for financial risk management.

## 1. Introduction

Tail risks, which can be quantified by risk measurements such as quantile, expectile, and entropic value at risk [1], highlight the potential for serious losses that could affect investors, financial institutions, and the overall stability of financial markets, making their measurement critical in the financial fields. Understanding and managing tail risk helps mitigate adverse consequences and maintain financial resilience. A voluminous literature provides econometric tools to measure tail risk ([2,3,4,5] and references therein). However, the presence of price-limit trading policies in certain markets complicates the accurate depiction of tail risk, as these constraints may distort the manifestation of tail risk. Price limits, widely endorsed across global stock and futures exchanges, serve as a safeguard procedure for investors and a deterrent against market manipulation [6,7,8]. By imposing restrictions on daily price fluctuations, these policies challenge the applicability of existing tail risk measures, typically designed for unrestricted markets. Consequently, this scenario urgently calls for tailored approaches in modeling tail risk within markets subject to price limits.

**Figure 1:**
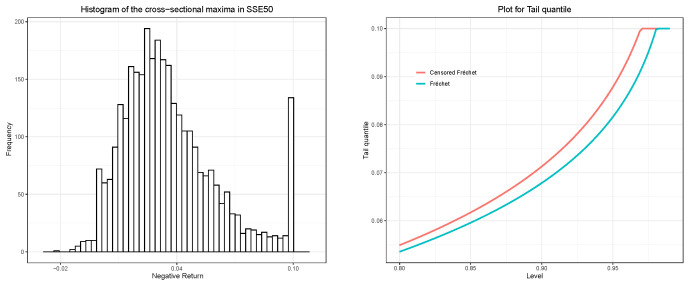
The left panel shows the histogram of the cross-sectional maxima of the negative daily returns of stocks in the SSE50 index for the period from 4 January 2005 to 30 December 2022; and the right panel shows the tail quantiles estimated by Fréchet distribution and censored Fréchet distribution, respectively.

The challenges posed by the price-limit policy are twofold. From an econometric point of view, the dispute on the pros and cons of the price-limit trading policy lasts for ages. Some argue that the price-limit policy may lead to ineffectiveness or even destructive market behavior [9,10,11], while others believe that a price-limited policy can reduce market manipulation risk and improve market efficiency [12,13,14]. The disorderly effectiveness of this policy makes it challenging to quantify its impact on tail risk. From a statistical point of view, this policy results in the censoring of observations, and ignoring such censoring could cause substantial bias and size distortion in measuring tail risk, even if the censored probability is tiny. The potential biases also occur in modeling the volatility of returns with price limits. To handle this case, Wei [15] develops a censored-GARCH model to recognize the unobservable feature of price-limited data and Hsieh and Yang [16] subsequently propose a censored stochastic volatility approach based on the censored-GARCH model to further improve the computational efficiency. However, both censored approaches rely on the algebraic relationship between the observed and latent returns (e.g., Equations (2)–(4) in [15]), which breaks down when dealing with the market’s aggregated information, such as tail risk, since it is jointly determined by multiple stocks. In addition, we present a simple analysis of the SSE50 (the SSE50 is a value-weighted price index that represents the performance of the top 50 firms listed on the Shanghai Stock Exchange, selected based on their market capitalization, liquidity, and other criteria) in Figure 1 to further illustrate the damage in measuring the tail risk caused by ignoring the censoring nature. The histogram in the left panel reveals a notable probabilistic stacking of observations triggered by the price limit, whereas the right panel demonstrates that the uncensored fitting method leads to an underestimation of tail risk (quantile).

In the domain of tail risk assessment, Extreme Value Theory (EVT; [17]) stands out as a potent instrument. This theory encompasses two principal methods: one approach is fitting the maximum observations using the generalized extreme value distribution (GEV) and is commonly referred to as the Maxima-GEV or Block Maxima (BM) method (e.g., [18]); another approach involves the Peak-over-Threshold (POT) method [19], which employs the Generalized Pareto Distribution (GPD) to approximate the conditional behavior of random variables that exceed specific high thresholds. Several studies have extensively explored the implications of EVT in assessing tail risk within the context of price limits. Oh et al. [20] assume that the conditional tail distribution of extreme returns obeys a power law and obtain an inferred estimation of tail risk under price limits. Subsequently, Ji et al. [21] introduce a general framework of the self-exciting point process with the truncated generalized Pareto distribution to measure the extreme risks in the price-limited stock markets. Nevertheless, the former ignore the censored nature of extreme returns when estimating the tail index, a critical aspect of accurate tail risk estimation. The latter use a truncated distribution rather than a censored structure to self-adapt to the price limits, which prevents this approach from modeling the latent return and results in their risk measurements continuing to be constrained by price limits, lacking sensitivity to extreme risk events. In addition, both methods ignore a dynamic treatment for the tail index, which has been demonstrated to be necessary by Massacci [22], Zhao et al. [23], and Shen et al. [24], etc. These studies have revealed substantial evidence that the tail risk in financial markets without price limits exhibits significant dynamics over time. Intuitively, these dynamic features of tail risk would also be present in price-limited markets.

For a deeper understanding of tail risk dynamics in price-limited markets, this paper focuses on modeling the time-varying tail features when observations beyond some threshold are censored. We propose a novel censored autoregressive conditional Fréchet model, which accommodates the censoring, heavy-tailed, volatility clustering, and extreme event clustering nature of financial data. The CAcF model incorporates a flexible observation-driven time evolution scheme of the parameters $\sigma_{t}$ (volatility index) and $\alpha_{t}$ (tail index) of a Fréchet (Type-II GEV) distribution, and the censoring feature into the modeling, allowing for a more explicit exploration of the time-varying tail behavior in price-limited equity markets. Moreover, we employ three typical observation-driven functions to decompose the tail risk from varying risk preference perspectives. (Risk preference is a pivotal factor in economic behavior, directly influencing the choice and behavior of investors in risk investment decisions [25,26,27]).

To empirically illustrate our findings, we utilize stock data from companies included in the SSE50, CSI300 (the CSI300 is a broader index that encompasses the top 300 firms listed on the Shanghai Stock Exchange and Shenzhen Stock Exchange), and TW50 (the TW50 is a market capitalization-weighted stock index developed by the Taiwan Stock Exchange in cooperation with the Financial Times and Stock Exchange (FTSE), which comprises the 50 companies with the largest market capitalization listed on the Taiwan Stock Exchange). In terms of our proposed model, we offer a maximum likelihood estimation (MLE) procedure for model estimation. To quantify tail risk, we adopt the entropic value at risk, which incorporates self-information via entropy and allows for a more flexible and robust representation of risk. We have also derived closed-form expressions for entropic value at risk and censored probability within this framework, providing a convenient approach for out-of-sample prediction. The empirical estimation results demonstrate that the CAcF model can effectively monitor time-varying behaviors of tail risk and provide satisfactory forecasting performance. This suggests its potential value in warning against financial tail risks. In addition, the tail risk of price-limited stock markets is significantly underestimated when censoring is not taken into account. Moreover, our analysis results show that the CAcF-type models with different risk preferences yield varied interpretations of risk. Specifically, risk-preferred investors perceive that hitting limit-down will reduce the potential risk at the next moment, whereas risk-averse investors interpret it conversely. These findings align with the principles of investment psychology and market dynamics. Finally, we study the impact of widening price limits by comparing the performance of the tail risks of TW50 over periods with different price limits. The evidence shows that widening price limits would lead to a decrease in the incidence of extreme events (hitting limit-down), but a significant increase in tail risk.

This paper provides a twofold contribution to the growing literature on tail risk measurement in financial markets. From a statistical modeling point of view, we propose a dynamic tail risk model for price-limited financial markets. The CAcF model incorporates a flexible observation-driven time evolution scheme for the key parameters and accommodates many important empirical characteristics of financial data. We demonstrate that the CAcF model can be derived from a general factor model, which ensures that the dynamic model is theoretically feasible. From an econometric point of view, the CAcF model offers a new perspective to illustrate tail risk dynamics when price limits exist in financial markets. Real applications show that tail risk is seriously underestimated when the price-limited constraint is ignored. Moreover, this study provides valuable insights for policymakers to develop more effective price-limited policies from a risk management perspective.

The rest of this paper is structured as follows. Section 2 introduces the framework of the CAcF model and derives a maximum likelihood estimation procedure. Section 3 presents the empirical results and analysis. Finally, we conclude the paper in Section 4.

## 2. Methodology

### 2.1. Model Specification

Let $\left\{Q_{t}\right\}$ denote the maximal time series representing the cross-sectional maximum of the negative daily return $X_{it}$ of the stock prices of *N* companies occurring on day *t*, i.e., $Q_{t}=\max_{1\leq i\leq N}X_{it}$. Essentially, $Q_{t}$ can offer insight into the potential tail risk of a specific stock market. However, in a price-limited stock market, the econometrician observes that $\left\{Q_{t}\right\}$ is censored by the following mechanism:

(1) $$Q_{t}=D_{t}M+\left(1-D_{t}\right)Q_{t}^{*},$$

where $D_{t}=I\left[Q_{t}^{*}>M\right]$, *M* is a constant censoring threshold determined by the price limit, $Q_{t}^{*}$ is the latent return but unobserved, and I[·] denotes the indicator function.

**Remark 1.**  *Constrained by the price-limit policy, the observed maximal negative return $Q_{t}$ should be interval censored. However, it is almost impossible for all the stocks in a specific stock market to simultaneously hit the limit-up, which suggests that left-censoring is almost infeasible for $Q_{t}$. This allows us to relax the assumption on $Q_{t}$ from interval-censored to right-censored as defined in* (1).

According to the Fisher–Tippett–Gnedenko theorem [28,29], $Q_{t}$ can typically be modeled, under an independent and identically distributed (i.i.d.) assumption, with a truncated GEV distribution. Nevertheless, the classical approach neglects the time dependence among $\left\{Q_{t}\right\}$ and other characteristics, such as censored and heteroskedasticity. To address time-varying behaviors and censored nature of $Q_{t}$, building upon the dynamic GEV framework (autoregressive conditional Fréchet model, AcF) proposed by Zhao et al. [23], we now introduce the following censored autoregressive conditional Fréchet (CAcF) model:

(2) $$\begin{array}{cc} Q_{t}^{*} & =\mu +\sigma_{t}Y_{t}^{-1/\alpha_{t}},\\ Q_{t} & =D_{t}M+\left(1-D_{t}\right)Q_{t}^{*},\\ \log\sigma_{t} & =\beta_{0}+\beta_{1}\log\sigma_{t-1}+\left(\beta_{2}+\beta_{2}^{*}D_{t-1}\right)G_{1}\left(Q_{t-1}\right),\\ \log\alpha_{t} & =\gamma_{0}+\gamma_{1}\log\alpha_{t-1}-\left(\gamma_{2}+\gamma_{2}^{*}D_{t-1}\right)G_{2}\left(Q_{t-1}\right), \end{array}$$

where $0\leq \beta_{1}\neq \gamma_{1}<1$, $\beta_{2}^{*},\gamma_{2}^{*}\in R$, and $\beta_{2},\gamma_{2}>0$. Further, $\left\{Y_{t}\right\}$ is a sequence of i.i.d. unit Fréchet random variables and $\left(\mu ,\sigma_{t},\alpha_{t}\right)\in F_{t-1}=\sigma \left(Q_{t-1},D_{t-1},Q_{t-2},D_{t-2},\ldots \right)$ represents the location parameter, scale parameter, and shape parameter. The two terms $G_{1}\left(\cdot \right)$ and $G_{2}\left(\cdot \right)$ are the observation-driven functions for $\left\{\log\sigma_{t}\right\}$ and $\left\{\log\alpha_{t}\right\}$, and both are assumed to be monotonically increasing functions of $Q_{t-1}$. Combined with the autoregressive scheme for $\left\{\log\sigma_{t}\right\}$ and $\left\{\log\alpha_{t}\right\}$, this setting for the observation-driven functions ensures that the distribution of $Q_{t}$ has a larger scale (larger $\sigma_{t}$) and a heavier tail (smaller $\alpha_{t}$) than that of $Q_{t-1}$. In other words, this setting offers a joint modeling of both volatility clustering for $\left\{\sigma_{t}\right\}$ process and extreme event clustering for $\left\{\alpha_{t}\right\}$ process, resulting in a larger tail risk of $Q_{t}$ when an extreme event occurs at time t−1 (large $Q_{t-1}$).

**Remark 2.**  *In contrast to the conventional AcF model, the threshold M imposes an upper limit on the observable maximum sequence $\left\{Q_{t}\right\}$. The CAcF model degenerates to the AcF model when M=∞. Obviously, the CAcF model has a wider range of applications, including financial time series with price limits.*

**Remark 3.**  Similar to the TGARCH model [30], we insert a threshold structure, $\beta_{2}^{*}D_{t-1}$ ($\gamma_{2}^{*}D_{t-1}$), to compensate for the shock of hitting limit-down at time t−1 on the tail risk of $Q_{t}$. The compensation coefficient $\beta_{2}^{*}$ (as well as $\gamma_{2}^{*}$) represents the degree of risk compensation associated with hitting limit-down. A positive value for $\beta_{2}^{*}$ (as well as $\gamma_{2}^{*}$) suggests that hitting limit-down at the previous moment intensifies the risk clustering effect. In contrast, a negative value suggests a weakening of the risk clustering effect.

Notice that the boundedness of $Q_{t}$ allows us to relax the boundedness assumption for $G_{1}\left(\cdot \right)$ and $G_{2}\left(\cdot \right)$ in Zhao et al. [23], thus providing a wider range of choices for $G_{1}\left(\cdot \right)$ and $G_{2}\left(\cdot \right)$ compared to AcF model. Drawing on the relationships between risk preference and the convexity–concavity of the utility function in Expected Utility Theory (in this theory, the utility function whose expected value is maximized is convex for a risk-loving agent, linear for a risk-neutral agent, and concave for a risk-averse agent; see more details in Chapter 6 of Mas-Colell et al. [31]), we consider three types of observation-driven functions in the following:

- **Linear:** $G_{1}\left(z\right)=G_{2}\left(z\right)=z$.
- **Square:** $G_{1}\left(z\right)=G_{2}\left(z\right)=\mathrm{sgn}\left(z\right)z^{2}$, where sgn(·) is a sign function.
- **Exponential:** $G_{1}\left(z\right)=-\exp\left(-\beta_{3}z\right)$ and $G_{2}\left(z\right)=-\exp\left(-\gamma_{3}z\right)$, where $\beta_{3},\gamma_{3}>0$.

The CAcF models corresponding to the three different functions mentioned above are denoted as CAcF-L (Linear), CAcF-S (Square), and CAcF-E (Exponential), respectively. The main diversity among these three models is that the convexity–concavity of their observation-driven functions differs when faced with loss ($Q_{t-1}>0$). This diversity allows the three models to analyze the impact of historical information on tail risk from different risk preference perspectives. First, the CAcF-L model implies that the impact of the increase in loss is marginally constant. Thus, the CAcF-L model is suitable for those investors or regulators with a risk-neutral preference. Second, the CAcF-S model assumes that an incremental loss can result in a rapid escalation of unease or pessimism about future risk, displaying a high sensitivity to losses. This characteristic is typical of risk-averse individuals. Finally, the CAcF-E model supposes that the impact of the increase in loss is marginally decreasing, which represents the attitude of risk lovers.

Together with the threshold structure in (2), these three types of observation-driven functions allow us to analyze the decisions of investors with different risk perspectives when hitting limit-down. For example, if the compensation coefficients are negative ($\beta_{2}^{*},\gamma_{2}^{*}<0$) in the CAcF-E model, this scenario means that a risk lover may regard hitting limit-down as a signal to buy stocks. A potential explanation is that risk lovers believe that the magnet effect of hitting limit-down leads to an arbitrage opportunity in the stock market. This interpretability of the CAcF model makes it more competitive in analyzing the tail risks of price-limited financial markets.

**Remark 4.**  *Note that the relationships between risk preference and the convexity–concavity of the observation-driven functions in CAcF models are the reverse of those in Expected Utility Theory. This reversal occurs because the CAcF models focus on losses while Expected Utility Theory concentrates on returns.*

**Remark 5.**  *Although the three CAcF models employ different observation-driven functions, their recovered $\left\{\sigma_{t}\right\}$ and $\left\{\alpha_{t}\right\}$ processes are highly similar. This is because the recovered $\left\{\sigma_{t}\right\}$ and $\left\{\alpha_{t}\right\}$ processes by different CAcF models are constrained by the same autoregression structure and the same log-likelihood function. Moreover, we verify this in Section 3. Consequently, the three CAcF models have a consensus on the estimation of tail risk.*

### 2.2. CAcF Model under a Factor Model Framework

In this subsection, we show that the CAcF model can be derived from the limiting form of maximum $Q_{t}$ under a latent general factor model framework. Assume ${\left\{x_{it}\right\}}_{i=1}^{N}$ is subject to a latent general factor model,

(3) $$\begin{array}{cc} X_{it} & =\left\{\begin{array}{cc} M,\; & X_{it}^{*}\geq M,\\ X_{it}^{*},\; & -M<X_{it}^{*}<M,\\ -M,\; & X_{it}^{*}\leq -M, \end{array}\right. \end{array}$$

(4) $$\begin{array}{cc} X_{it}^{*} & =f_{i}\left(Z_{1t},Z_{2t},\ldots ,Z_{dt}\right)+\sigma_{it}\varepsilon_{it}, \end{array}$$

where ${\left\{X_{it}\right\}}_{i=1}^{p}$ and ${\left\{X_{it}^{*}\right\}}_{i=1}^{p}$ are observed and unobserved latent negative returns of company *i* at time *t*, respectively, *M* is a constant censoring threshold determined by the price limit, $\left\{Z_{1t},Z_{2t},\ldots ,Z_{dt}\right\}$ consist of observed and unobserved factors, ${\left\{\varepsilon_{it}\right\}}_{i=1}^{N}$ are i.i.d. noises that are independent of the factors ${\left\{Z_{it}\right\}}_{i=1}^{d}$ and ${\left\{\sigma_{it}\right\}}_{i=1}^{N}\in F_{t-1}$ are the conditional volatilities of ${\left\{X_{it}^{*}\right\}}_{i=1}^{N}.$ The function $f_{i}:R^{d}\to R$ is a Borel function.

Factor model has been widely used for modeling asset returns [32,33,34]. The general factor model (4) can handle common properties among $X_{it}$, such as heterogeneous volatilities and cross-sectional dependence. Together with (3), such a factor structure can well model asset returns in the price-limited market. To incorporate another important characteristic, heavy-tailedness, of many financial time series, we assume that the random noises ${\left\{\varepsilon_{it}\right\}}_{i=1}^{N}$ are random variables in the Domain of Attraction of Fréchet distribution [35]. Distributions in the Domain of Attraction of Fréchet distribution include a broad class of random variables such as Cauchy, Lévy, Pareto, and *t* distributions. A specific definition is described in Appendix B.

Further, we consider the following Assumption 1.

**Assumption 1.** 

*(a)*  *Under a dynamic model, the tail index $\alpha_{it}$ of $\varepsilon_{it}$ evolves through time according to certain dynamics and $\alpha_{t}\in F_{t-1}$. In addition, ${\left\{\varepsilon_{it}\right\}}_{i=1}^{N}$ are i.i.d. random variables in the Domain of Attraction of Fréchet distribution with a tail index $\alpha_{t}$.*
*(b)* 
$\sup_{1\leq N<\infty }\sup_{1\leq i\leq N}\left|f_{i}\left(Z_{1t},Z_{2t},\ldots ,Z_{dt}\right)\right|<\infty ,\;a.s.$

*(c)*  *$\lim_{N\to \infty }\sum_{i=1}^{N}\sigma_{it}^{\alpha_{t}}=\infty \;\mathrm{and}\;\lim_{N\to \infty }\sup_{1\leq i\leq N}{\left(\sum_{j=1}^{N}\sigma_{jt}^{\alpha_{t}}\right)}^{-1}\sigma_{it}^{\alpha_{t}}=0$.*

Assumption 1(a) commonly addresses the heavy-tailed nature of financial time series within factor models. Assumption 1(b) is a mild assumption about the boundedness of $f_{i}$. Assumption 1(c) means the magnitudes of conditional volatility $\sigma_{it}$ are comparable to each other and there is no single $X_{it}$ that dominates the total volatility.

**Proposition 1.**  *Denote $Q_{t}=\max_{1\leq i\leq N}X_{it}$, $a_{Nt}=0$, and $b_{Nt}={\left(\sum_{i=1}^{N}\sigma_{it}^{\alpha_{t}}\right)}^{1/\alpha_{t}}$. Under Assumptions 1 and given $F_{t-1}$, we have, as N→∞,*

$$\frac{Q_{t}-a_{Nt}}{b_{Nt}}\overset{d}{⟶}\max\left(\Psi_{\alpha_{t}},\left(M-a_{Nt}\right)/b_{Nt}\right),$$

*where $\Psi_{\alpha_{t}}$ is a Fréchet type random variable with tail index $\alpha_{t}$ and the cumulative distribution function of $\Psi_{\alpha_{t}}$ is $\exp\left(-x^{-\alpha_{t}}\right)$.*

The proof of Proposition 1 is presented in Appendix B. Proposition 1 shows that, under the framework of the general factor model and some mild conditions, the conditional distribution of $Q_{t}$ can be well approximated by a right-censored Fréchet distribution, which provides the rationality of the CAcF model.

### 2.3. Parameter Estimation

We denote all the parameters in the model by $\theta =\left(\mu ,\beta_{0},\beta_{1},\beta_{2},\beta_{2}^{*},\beta_{3},\gamma_{0},\gamma_{1},\gamma_{2},\gamma_{2}^{*},\gamma_{3}\right)$ and the constraints $\Theta =\left\{\theta |\mu ,\beta_{0},\gamma_{0},\beta_{2}^{*},\gamma_{2}^{*}\in R,0\leq \beta_{1},\gamma_{1}<1,\beta_{2},\beta_{3},\gamma_{2},\gamma_{3}>0\right\}$. Using model setting and conditional independence, we can write the log-likelihood function with censored observations ${\left\{Q_{t}\right\}}_{t=1}^{n}$ as

(5) $$\begin{array}{cc} L_{n}\left(\theta \right) & =\frac{1}{n}\sum_{t=1}^{n}\left[D_{t}\log P\left(Q_{t}^{*}>M|F_{t-1}\right)+\left(1-D_{t}\right)\log f_{Q_{t}^{*}}\left(Q_{t}|F_{t-1}\right)\right]\\ \; & =\frac{1}{n}\sum_{t=1}^{n}[D_{t}\log\left(1-\exp(-\sigma_{t}^{\alpha_{t}}{\left(M-\mu \right)}^{-\alpha_{t}})\right)+\left(1-D_{t}\right)[\log\alpha_{t}\\ \; & \;\;+\alpha_{t}\log\sigma_{t}-\left(\alpha_{t}+1\right)\log\left(Q_{t}-\mu \right)-\sigma_{t}^{\alpha_{t}}{\left(Q_{t}-\mu \right)}^{-\alpha_{t}}]], \end{array}$$

where $f_{Q_{t}^{*}}\left(\cdot |F_{t-1}\right)$ is the probability density function of $Q_{t}^{*}$ conditional on the past information $F_{t-1}$, and ${\left\{\sigma_{t},\alpha_{t}\right\}}_{t=1}^{n}$ can be obtained recursively through (2) with an initial value $\left(\sigma_{1},\alpha_{1}\right)$. Subsequently, the maximum likelihood estimator $\hat{\theta }$ can be obtained by iterative search.

**Remark 6.**  *In practice, we use the estimated $\left(\hat{\sigma },\hat{\alpha }\right)$ from the static censored Fréchet distribution with the threshold M as the initial value for $\left(\sigma_{1},\alpha_{1}\right)$. Since $0\leq \beta_{1},\gamma_{1}<1$, the influence of $\left(\sigma_{1},\alpha_{1}\right)$ on recovered $\left({\hat{\sigma }}_{t},{\hat{\alpha }}_{t}\right)$ decays exponentially as t increases. Hence, the impact of $\left(\sigma_{1},\alpha_{1}\right)$ on parameter estimation could be ignored with a sufficiently large sample size.*

### 2.4. Prediction of Maximum Negative Return, Entropic Value at Risk, and Censored Probability

In this subsection, we derive several closed-form expressions for tail risk prediction. First, we give the one-step forward recursive prediction of the maximum negative return $Q_{t}$ as follows:

$$\begin{array}{c} {\hat{Q}}_{t}=E\left[Q_{t}|F_{t-1}\right]=M+\sigma_{t}\Gamma \left(1-1/\alpha_{t},\sigma_{t}^{\alpha_{t}}{\left(M-\mu \right)}^{-\alpha_{t}}\right)-\left(M-\mu \right)\exp\left(-\sigma_{t}^{\alpha_{t}}{\left(M-\mu \right)}^{-\alpha_{t}}\right), \end{array}$$

where the upper incomplete gamma function $\Gamma \left(a,b\right)=\int_{b}^{\infty }x^{a-1}\exp\left(-x\right)dx$.

Second, we employ the entropic value at risk (EVaR) based on Rényi entropy, which is proposed by Pichler and Schlotter [1], as a statistical metric to quantify the level of tail risk associated with an investment portfolio, security, or company. Specifically, we adopt the following definition:

**Definition 1**  (Pichler and Schlotter [1]). *The entropic value at risk ${\mathrm{EVaR}}_{\tau }^{p}\left(X\right)$ of order p∈R at confidence level τ∈[0,1) and $X\in L^{p}$ based on Rényi entropy is*

$${\mathrm{EVaR}}_{\tau }^{p}\left(X\right):=\sup\left\{EXZ:Z\geq 0,EZ=1\;\mathrm{and}\;H_{q}\left(Z\right)\leq \log\frac{1}{1-\tau }\right\},$$

*where $\frac{1}{p}+\frac{1}{q}=1$ and $H_{q}\left(Z\right)$ is the Rényi entropy of order q∈R of a random variable Z, i.e.,*

$$H_{q}\left(Z\right):=\left\{\begin{array}{cc} -\log P(Z>0) & \;\mathrm{if}\;q=0,\\ Z\log Z & \;\mathrm{if}\;q=1,\\ {\log∥Z∥}_{\infty } & \;\mathrm{if}\;q=\infty ,\\ \frac{1}{q-1}\log EZ^{q} & \;\mathrm{else}.\; \end{array}\right.$$

Since the CAcF-type models are based on the Fréchet distribution, whose *p*-th moment exists if its tail index α>p (i.e., a Fréchet random variable $X\in L^{p}$ if and only if its tail index α>p), and the estimated tail index $\alpha_{t}$ ranges from 2 to 12 in our empirical analysis, we mainly focus on the entropic value at risk of order p=1 at confidence level τ (${\mathrm{EVaR}}_{\tau }^{1}$) in the following content. This has another potential benefit in that ${\mathrm{EVaR}}_{\tau }^{1}$ is a coherent risk measure (see more details in [36]). According to Theorem 12 of Pichler and Schlotter [1], ${\mathrm{EVaR}}_{\tau }^{1}$ has the dual representation

$${\mathrm{EVaR}}_{\tau }^{1}\left(X\right)=\underset{c\in R}{\inf}\left\{c+\frac{1}{1-\tau }\cdot E\left(\max(X-c,0)\right)\right\}.$$

Note that ${\mathrm{EVaR}}_{\tau }^{1}$ is equal to the conditional value at risk (CVaR, [36,37]), also known as the expected shortfall (ES). Then, the one-step forward predictable conditional ${\mathrm{EVaR}}_{\tau }^{1}$ of $Q_{t}^{*}$ and $Q_{t}$ are

$${\mathrm{EVaR}}_{\tau ,Q_{t}^{*}|F_{t-1}}^{1}=\mu +\frac{\sigma_{t}}{1-\tau }\gamma \left(1-1/\alpha_{t},-\log\tau \right),$$

where the lower incomplete gamma function $\gamma \left(a,b\right)=\int_{0}^{b}x^{a-1}\exp\left(-x\right)dx$. We call ${\mathrm{EVaR}}_{1-\tau ,Q_{t}^{*}|F_{t-1}}^{1}$ *uncensored EVaR* in the following content. Both the conditional EVaRs of $Q_{t}^{*}$ and $Q_{t}$ reflect the tail risk of a specific price-limited market. However, the conditional EVaR of $Q_{t}$, limited by its boundedness, cannot reflect the severity of the risks suffered by a specific price-limited market in a financial crisis. Instead, the uncensored EVaR is a more suitable risk measurement for price-limited markets.

Finally, an alternative indicator that can be used to monitor the tail risk of a price-limited market is the censored probability, whose one-step forward prediction is given as follows:

$${\hat{P}}_{t}=P\left(Q_{t}^{*}>M|F_{t-1}\right)=1-\exp\left(-\sigma_{t}^{\alpha_{t}}{\left(M-\mu \right)}^{-\alpha_{t}}\right).$$

## 3. Empirical Analysis

### 3.1. Chinese Mainland Stock Market

The implementation of the price-limit policy in the stock markets of mainland China on 16 December 1996 necessitated a ±10% limit on the fluctuation of stock prices relative to their previous closing price. To elucidate the dynamics of tail risk within the Chinese equity market, this study applies the CAcF models to examine the cross-sectional maximum of negative daily returns among stocks listed on two pivotal Chinese stock indices: the SSE50 and the CSI300. These indices are not only paramount but also the most frequently cited barometers of Chinese financial market health and are considered reflective of the broader sentiment pervading the Chinese equity landscape. The empirical inquiry spans from 4 January 2005 to 30 December 2022, aggregating 4374 data points. For model calibration, the study delineates the period between 4 January 2005 and 30 December 2020, deploying the subsequent data from 4 January 2021 to 30 December 2022 for out-of-sample predictions.

Note that the price limit was relaxed to ±20% on the Science and Technology Innovation Board and Second Board starting 13 June 2019 and 24 August 2020, respectively. For the homogeneity of data, this analysis excludes stocks in SSE50 and CSI300 that are not part of the Main Board, thereby maintaining adherence to the ±10% price limit. It is imperative to note that the proportion and temporal span of stocks subjected to the ±20% threshold within the SSE50 and CSI300 only account for a small fraction. Consequently, the exclusion of these data subsets does not materially impede the assessment of tail risk associated with the indices.

#### 3.1.1. Fitting Results for SSE50 and CSI300

We employed the CAcF models to fit the in-sample data and conducted a comparative analysis with the AcF model, which ignores the censored nature. The results of parameter estimation are presented in Table 1. This comparison yielded several noteworthy insights. Specifically, analysis of the estimated location parameter, μ, reveals that, for both the SSE50 and CSI300 datasets, all three variants of the CAcF model exhibit lower absolute values in the estimated location parameters compared to the AcF model, with correspondingly reduced standard deviations. Furthermore, it is observed that the location parameter μ estimated by the AcF model significantly deviates from the observed value range. This evidence suggests that neglecting the censored characteristics of the data introduces substantial bias and size distortion to the location parameter estimation, potentially leading to inaccurate tail risk assessment when censored observations are modeled using the AcF approach.

Subsequently, we focus on analyzing the estimated autoregressive coefficients $\beta_{1},\gamma_{1}$ and the estimated compensation coefficients $\beta_{2}^{*},\gamma_{2}^{*}$ for the volatility index process $\left\{\sigma_{t}\right\}$ and the tail index process $\left\{\alpha_{t}\right\}$. The remaining coefficients only determine the movement range of $\left\{\sigma_{t}\right\}$ and $\left\{\alpha_{t}\right\}$ without other particular meanings. For the SSE50 dataset, all the estimated autoregressive coefficients $\beta_{1}$ and $\gamma_{1}$ range from 0.83 to 0.88, indicating a strong persistence in both the $\left\{\sigma_{t}\right\}$ process and the $\left\{\alpha_{t}\right\}$ process while, for the CSI300 dataset, all the estimated autoregressive coefficients are uniformly smaller than those for the SSE50 dataset, which suggests that there is a stronger observation-driven latent factor. This is understandable because the CSI300 index includes more constituent stocks than the SSE50 index, making the observation process $\left\{Q_{t}\right\}$ contain more latent information. Recall model (2); the compensation coefficients $\beta_{2}^{*}$ and $\gamma_{2}^{*}$ represent the degree of risk compensation associated with hitting limit-down, and reflect the decisions of investors with different risk perspectives when hitting limit-down. For both the SSE50 dataset and the CSI300 dataset, the compensation coefficients of the CAcF-E model are negative, while the compensation coefficients of the CAcF-L and CAcF-S models are positive. This result indicates that, in the Chinese mainland stock market, risk lovers regard hitting limit-down as a signal to buy stocks, while risk-neutral or risk-averse agents regard hitting limit-down as a signal that triggers further deterioration of stock markets. We can also see from Table 1 that the significance of the CAcF model coefficients is significantly better than that of the AcF model coefficients. This result is also strong evidence that the CAcF models are more suitable for price-limited markets.

**Figure 2:**
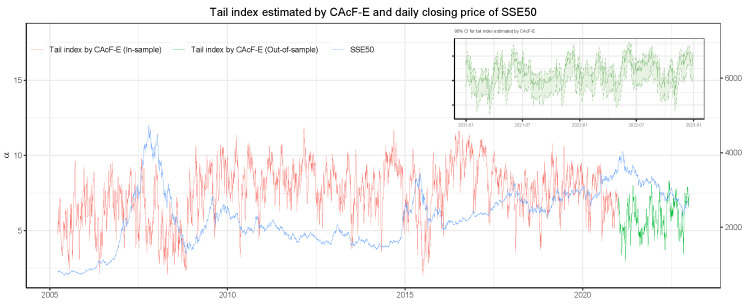
The figure shows the estimated tail index $\left\{{\hat{\alpha }}_{t}\right\}$ by CAcF-E (red line) and the corresponding predicted tail index (green line). The blue line represents the closing price of SSE50. The top-right subfigure presents the 90% confidence interval for the predicted tail index.

**Figure 3:**
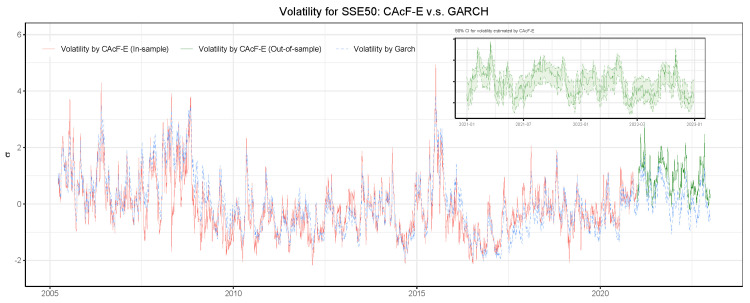
Estimated volatility: CAcF v.s. GARCH. The red solid line is the estimated $\left\{{\hat{\sigma }}_{t}\right\}$ by CAcF-E, the green solid line is the corresponding predicted value, and the blue dashed line is the estimated volatility by GARCH(1,1) model. All series are standardized to be zero mean and unit variance for comparison. The top-right subfigure presents the 90% confidence interval for the predicted volatility.

Furthermore, we present the CAcF-E modeling results for the SSE50 in detail, where Figure 2 and Figure 3 show the estimation results of the tail index $\left\{{\hat{\alpha }}_{t}\right\}$ and the volatility index $\left\{{\hat{\sigma }}_{t}\right\}$, respectively. More results for other models and the CSI300 dataset are given in Appendix D. In Figure 2, the red line represents the estimated $\left\{{\hat{\alpha }}_{t}\right\}$ and the green line represents the one-step prediction of the tail index base on historical information $F_{t-1}$. A smaller ${\hat{\alpha }}_{t}$ indicates greater tail risk. Overall, the estimated tail index of the CAcF-E model is roughly in the range of 2 to 12. The two periods in which the tail index is consistently smaller ($\alpha_{t}<4$) coincide with the Subprime Crisis and the Chinese stock market crash in 2015. There is a significant correlation between the upward and downward trends in the closing price series of SSE50 and the estimated tail index, making ${\hat{\alpha }}_{t}$ a valid indicator of the tail risk of the underlying market, i.e., the market stability index. In addition, before the SSE50 index declined significantly during the 2015 Chinese stock market crash, the estimated tail index based on the CAcF-E model had already begun to fall suddenly and sharply, providing an early warning of significant risks in the market. The performance of the tail index estimated via the CAcF-E model is consistent with those empirical findings of Massacci [22] and Zhao et al. [23] in markets without a price-limit policy. Moreover, the top-right subfigure presents the point-wise 90% confidence interval for the tail index based on historical information $F_{t-2}$. (The closed-form expressions for confidence intervals of ${\hat{\alpha }}_{t}$ and ${\hat{\sigma }}_{t}$ are shown in Appendix C.) Instead of using a one-step point forecast, investors can use the lower bound of the confidence interval of the tail index as an earlier risk warning. Analogously, volatility risk in the market is also of great concern to investors. Figure 3 represents the estimated $\left\{{\hat{\sigma }}_{t}\right\}$ and the one-step prediction of the volatility together with the point-wise 90% confidence interval in the top-right subfigure. For comparison, we also fit a GARCH(1,1) model for the cross-sectional maxima of the negative daily returns of the stocks in SSE50 and plot the standardized daily volatility estimated by the GARCH model in Figure 3 (blue dashed line). The two series are very close, suggesting that CAcF’s dynamic scaling parameter $\sigma_{t}$ (including its forecasts) accurately measures market volatility.

To verify the discussion in Remark 5, we employ two measures of sequence similarity, namely Cosine Distance (CosD) and mean Euclidean Distance (MED), defined as follows

$$\mathrm{CosD}\left(a_{t},b_{t}\right)=1-\sum a_{t}b_{t}/\sqrt{\sum a_{t}^{2}\sum b_{t}^{2}}\;\mathrm{and}\;\mathrm{MED}\left(a_{t},b_{t}\right)=\frac{1}{n}\sqrt{\sum {\left(a_{t}-b_{t}\right)}^{2}},$$

where $\left\{a_{t},1\leq t\leq n\right\}$ and $\left\{b_{t},1\leq t\leq n\right\}$ denote two different estimated dynamic parameter sequences. A pairwise comparison of the estimates of the dynamic scale parameter and the shape parameter for the three different CAcF models is presented in Table 2. It is observed that the two index sequences obtained from the different CAcF-type models are highly similar, regardless of whether it is an angular measure (CosD) or a distance measure (MED). This result suggests that the three CAcF models only decompose the $\left\{\sigma_{t}\right\}$ process and the $\left\{\alpha_{t}\right\}$ process from different risk preference perspectives through different observation-driven functions, but these three models are consistent in their estimates of the $\left\{\sigma_{t}\right\}$ process and the $\left\{\alpha_{t}\right\}$ process. In addition, combining the close estimated location parameters of the three CAcF models, this result further indicates that the three CAcF models have a consensus on the estimation of tail risk.

#### 3.1.2. Tail Risk Analysis and Forecasting Performance

**Figure 4:**
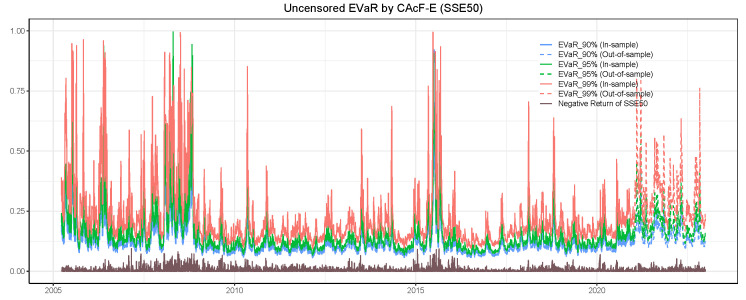
The figure shows the in-sample and out-of-sample predicted results for CAcF-based entropic value at risk (VaR) of the $Q_{t}^{*}$ in SSE50. The brown line indicates the negative daily returns of the SSE50 index (the positive daily returns of the SSE50 index are cut off to reflect the downward risk better).

**Figure 5:**
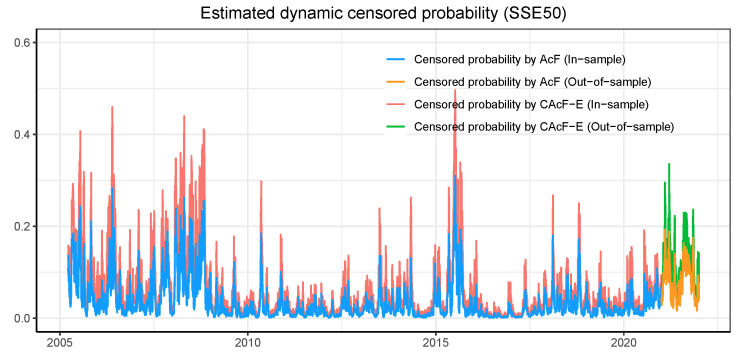
The figure shows the in-sample and out-of-sample predicted results for CAcF-based censored probability of the cross-sectional maxima of the negative daily returns of the stocks in SSE50.

In this subsection, we apply the CAcF models to assess time-varying tail risk. Figure 4 shows both in-sample and out-of-sample forecasts of CAcF-based entropic value at risk with order p=1 of the $Q_{t}^{*}$ series in SSE50 at the 90%, 95%, and 99% confidence levels (solid lines denote in-sample and dashed lines denote out-of-sample). Notably, CAcF-based EVaR shows its sensitivity to market turbulence and reaches high levels around 2007–2008 and 2015, which is consistent with the actual events of the Subprime Crisis and the Chinese stock market crash in 2015. The strength of its fluctuation is also consistent with the brown line below indicating the negative daily return of the SSE50 index. Figure 5 elucidates the probability of actual observations being censored—specifically, the likelihood of encountering the limit-down scenario, as inferred from the latent maximum series $Q_{t}^{*}$ distribution, which is derived from the parameters estimated by both the CAcF-E and AcF models. A significant takeaway from this analysis is the discernibly higher probability of breaching price limits when acknowledging the censored nature of the data. This observation intimates a more dire market scenario than might otherwise be perceived. The rationale behind this finding is intuitively straightforward: when the censored data characteristic is overlooked, a 10% decline is interpreted as a genuine market movement. However, this interpretation often overlooks the critical role of price-limit constraints, which, if absent, would likely result in declines exceeding 10%, thereby indicating a heightened risk scenario.

To illustrate the forecasting performance of the CAcF models, we employ three evaluation measures to evaluate the out-of-sample performance of recursive prediction from 4 January 2021 to 30 December 2022, and the results are detailed in Table 3. The three metrics include mean absolute error (MAE), mean absolute percentage error (MAPE), and mean censored probability (MCP), defined as follows,

$$\mathrm{MAE}=\frac{1}{n}\sum_{t}|{\hat{Q}}_{t}-Q_{t}|,\;\mathrm{MAPE}=\frac{1}{n}\sum_{t}|\left({\hat{Q}}_{t}-Q_{t}\right)/Q_{t}|,\;\mathrm{MCP}=\frac{1}{n}\sum_{t}\left|{\hat{P}}_{t}-D_{t}\right|,$$

where ${\hat{Q}}_{t}$ denotes the prediction of $Q_{t}$ and ${\hat{P}}_{t}=\hat{E}\left[D_{t}|F_{t-1}\right]$ denotes the probability of hitting limit-down calculated based on the distribution of $Q_{t}^{*}$ in model (2). In both the SSE50 or CSI300 datasets, all three CAcF models obtained smaller values of MAE, MAPE, and MCP, i.e., better predictive performance, than the benchmark AcF model. For example, for the out-of-sample prediction of $Q_{t}$, the MAPE of the CAcF-L model for CSI300 is 0.2706, which is 9% less than 0.2973 in the AcF model. Further, for the out-of-sample prediction of $D_{t}$, there is a 96% reduction. The comparison results demonstrate that the censored structure of CAcF models effectively improves the prediction performance.

### 3.2. Chinese Taiwan Stock Market

For the Taiwan stock market, the price-limit policy has been revised several times. The price limits were ±5% until January 1989,±7% from January 1989 to 30 May 2015, and ±10% thereafter. We collect the historical stock data from 4 January 2005 to 30 December 2022, with a total of 4431 observations. Since the sample period covers two different price-limit policies, we divide the data into two periods and fit them with the CAcF model. Period I is from 4 January 2005 to 30 May 2015, while period II is from 1 June 2015 to 30 December 2022. The price limits of these two periods are ±7% and ±10%, respectively.

In this part of our study, we are mainly concerned with two questions. First, we want to verify whether the patterns we observed in the stock market of mainland China are also present in other markets. Second, we are interested in how the relaxation of the price limits affects the tail risk of the stock market.

#### 3.2.1. Fitting Results for TW50

To accurately capture the variations in tail risk after the implementation of a new price limit in the Taiwan stock market, we do not split the sample data for out-of-sample validation in this study. The parameter estimation results for the TW50 index are described in Table 4. These findings echo the insights garnered from the analysis of the SSE50 and CSI300 indices, underscoring that employing the AcF model for censored observations could lead to an inaccurate assessment of tail risk. Notably, for both periods under review, the estimated autoregressive coefficients $\beta_{1}$ and $\gamma_{1}$ exhibit values ranging between 0.82 and 0.90, with minimal variance between the two periods. This observation suggests a notable consistency in the persistence of both the $\sigma_{t}$ and the $\alpha_{t}$ processes across the different periods. Regarding the compensation coefficients $\beta_{2}^{*},\gamma_{2}^{*}$, the estimated outcomes suggest that the investment behavior in the Taiwan stock market aligns with that observed within the Chinese mainland stock market, indicating a similar risk disposition among investors. Additionally, we compute the pairwise similarity of the dynamic scale parameter and the shape parameter across the three distinct CAcF models applied to the TW50 dataset. The outcomes of this analysis are presented in Table 5, offering yet another strong confirmation of Remark 5.

#### 3.2.2. Empirical Analysis on Widening Price Limits

**Figure 6:**
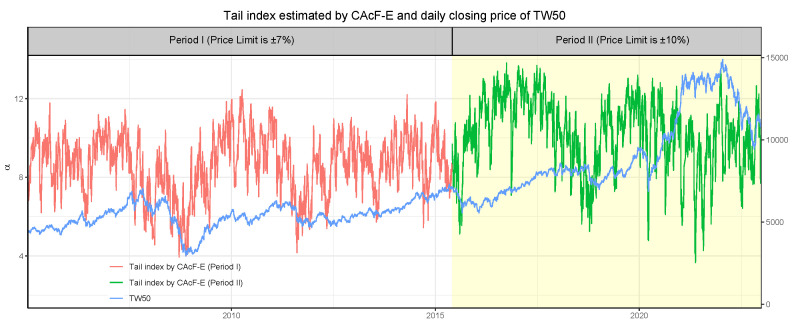
The estimated tail index $\left\{{\hat{\alpha }}_{t}\right\}$ of TW50 by CAcF-E, where the white area indicates results for Period I and the yellow area for Period II. The blue line illustrates the TW50’s closing price.

The adjustment of price limits in the Taiwan stock market offers an opportunity to examine the impact of broader price limits on tail risk. To this end, we reconstruct the tail index $\left\{\alpha_{t}\right\}$, volatility index $\left\{\sigma_{t}\right\}$, and tail risk processes for each of the two periods using the CAcF models. Figure 6 showcases the estimated tail index ${\hat{\alpha }}_{t}$ processes for both periods as derived through the CAcF-E model. Notably, there is no abrupt transition between the two tail index processes at the juncture of price-limit adjustment, with the estimated tail index for Period II exhibiting a broader range of movement compared to Period I. This suggests that the immediate probability of extreme events does not significantly alter following the introduction of new price limits. However, over the longer term, the likelihood of extreme events during tranquil market periods decreases, yet remains significant during turbulent times. This implies that, while broader price limits may reduce the frequency of hitting limit-down events, they do not mitigate tail risks during periods of market distress. This observation is partially supported by the fact that the incidence of limit-down events was 3.73% in Period I and reduced to 2.95% in Period II.

**Figure 7:**
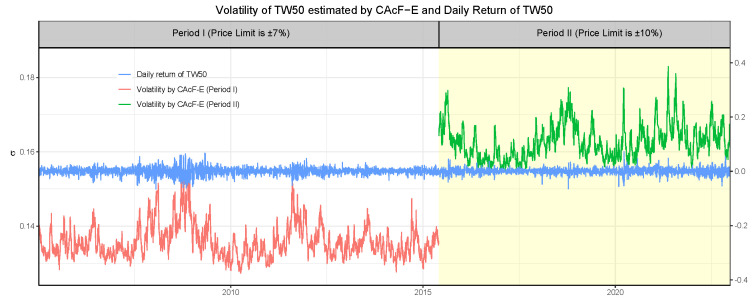
The estimated volatility index $\left\{{\hat{\sigma }}_{t}\right\}$ of TW50 by CAcF-E, where the white area indicates results for Period I and the yellow area for Period II. The blue line illustrates the TW50’s closing price.

The estimated volatility index processes ${\hat{\sigma }}_{t}$ are depicted in Figure 7, alongside the daily returns of the TW50 index. For both periods, the estimated volatility index process ${\hat{\sigma }}_{t}$ accurately captures the fluctuations in the TW50’s daily returns. However, unlike the tail index estimate ${\hat{\alpha }}_{t}$, there is a noticeable jump between the volatility indices for the two periods. This discrepancy indicates that the volatility of the cross-sectional maximum of negative daily returns for stocks listed on the TW50 index significantly improved after the expansion of price limits.

**Figure 8:**
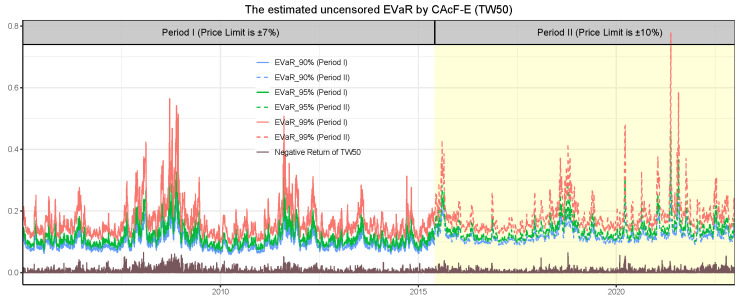
The CAcF-based entropic value at risk (EVaR) for the cross-sectional maximum, $Q_{t}^{*}$, of negative daily returns for TW50 stocks is depicted, with the white area illustrating results from Period I and the yellow area from Period II. The brown line represents the negative daily returns of the TW50 index, with positive daily returns excluded to better emphasize downward risk.

**Figure 9:**
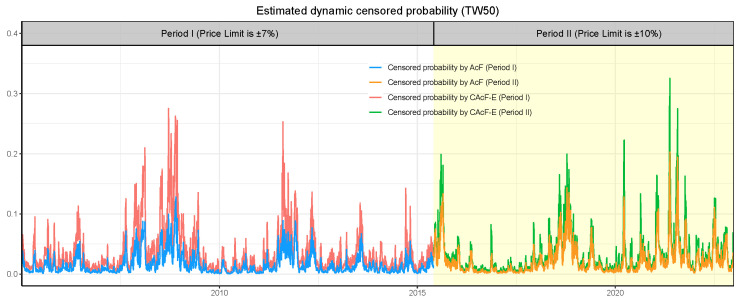
The censored probability derived from the CAcF-E model for the cross-sectional maxima of negative daily returns of TW50 stocks, with the white and yellow areas indicating the outcomes for Period I and Period II, respectively.

Figure 8 and Figure 9 depict the evolution of time-varying tail risk for the TW50 index, demonstrating the behavior of CAcF-based EVaR with order p=1 and the estimated probability of encountering censored events. These illustrations reveal pronounced increases in both the CAcF-based EVaR and the estimated censored probability during the 2007–2008 and 2020–2021 intervals, corresponding to the periods of the Subprime Mortgage Crisis and the COVID-19 pandemic. This correlation underscores the responsiveness of these risk metrics to market turmoil. Furthermore, the apex observed during the 2020–2021 period surpasses that of the 2007–2008 phase, suggesting that the widening of price limits not only enhances market volatility, as depicted in Figure 7, but also exacerbates tail risks. After relaxing price limits, the CAcF-based EVaR initially rises, then subsequently falls, reflecting a sophisticated market’s reaction to regulatory changes through the lens of price fluctuations. This pattern suggests that, while broader price limits can lead to initial instability, they may ultimately contribute to a more resilient market environment by allowing more freedom for price adjustments in response to market conditions.

## 4. Conclusions

This paper proposes a novel censored autoregressive conditional Fréchet model for analyzing the dynamic tail risk of financial markets constrained with price limits. Through empirically investigating the entropic value at risk of stock data from the Chinese mainland and Taiwan, we find that tail risk is seriously underestimated in price-limited stock markets when ignoring the censoring nature of data. We simultaneously decompose the tail risk from different risk preference perspectives via CAcF-type models with different observation-driven functions. The results offer guidelines and explanations for the decision-making for investors with diverse risk preferences dealing with risky events (hitting limit-down). Moreover, we explore the similarities and differences in the tail risk of the TW50 index over two periods with different price limits to study the impact of widening the price limit. The results suggest that widening the price limit would reduce the incidence of events of hitting limit-down; however, it also results in significant increases in the level of tail risk. The conclusions have important implications for policymakers in reconsidering market stabilization mechanisms. Finally, the out-of-sample performance of the CAcF model demonstrates its efficacy in forecasting tail risk and thus proves its potential for widespread application in tail risk monitoring.

However, the current study has some limitations, such as the lack of a way to analyze the risk relationship between multiple markets and the inability to include more lagged information. Therefore, in future applications, we would extend the CAcF model to the multivariate setting via a flexible extreme value copula to study the risk spillover effect among different stock markets. Another extended solution is to incorporate an ARMA structure for the time-varying parameters to utilize more historical information to enhance the forecasting performance of tail risk.

## Figures and Tables

**Table 1:** In-sample estimation results for SSE50 and CSI300.

	SSE50				CSI300			
	CAcF-L	CAcF-S	CAcF-E	AcF	CAcF-L	CAcF-S	CAcF-E	AcF
μ	−0.124 ***	−0.123 ***	−0.122 ***	−3.538	−0.182 ***	−0.180 ***	−0.179 ***	−5.864
	$\left(0.004\right)$	$\left(0.005\right)$	$\left(0.004\right)$	$\left(7.240\right)$	$\left(0.006\right)$	$\left(0.005\right)$	$\left(0.006\right)$	(4.938)
$\beta_{0}$	−0.297 ***	−0.309 ***	−0.206 ***	0.167	−0.514 ***	−0.497 ***	−0.415 ***	1.901 ***
	$\left(0.048\right)$	$\left(0.051\right)$	$\left(0.011\right)$	$\left(0.260\right)$	$\left(0.056\right)$	$\left(0.056\right)$	$\left(0.018\right)$	$\left(0.066\right)$
$\beta_{1}$	0.852 ***	0.842 ***	0.884 ***	0.869 ***	0.680 ***	0.683 ***	0.703 ***	0.636 ***
	$\left(0.023\right)$	$\left(0.026\right)$	$\left(0.006\right)$	$\left(0.018\right)$	$\left(0.035\right)$	$\left(0.036\right)$	$\left(0.013\right)$	$\left(0.031\right)$
$\beta_{2}$	0.420 ***	4.638 ***	0.035 ***	0.002	0.723 ***	5.596 ***	0.087 ***	1.257
	$\left(0.066\right)$	$\left(0.775\right)$	$\left(0.004\right)$	$\left(0.005\right)$	$\left(0.088\right)$	$\left(0.799\right)$	$\left(0.009\right)$	$\left(0.758\right)$
$\beta_{2}^{*}$	0.077	0.011	−0.194 ***	−	0.305 ***	2.139 **	−0.331 ***	−
	$\left(0.105\right)$	$\left(1.731\right)$	$\left(0.012\right)$	−	$\left(0.069\right)$	$\left(0.851\right)$	$\left(0.019\right)$	−
$\beta_{3}$	−	−	24.867 ***	13.577 ***	−	−	20.839 ***	0.025
	−	−	(2.556)	(5.236)	−	−	(1.476)	(0.091)
$\gamma_{0}$	0.447 ***	0.358 ***	−6.421 ***	−4.829 ***	0.839 ***	0.733 ***	−2.847 ***	0.932
	$\left(0.056\right)$	$\left(0.045\right)$	$\left(0.376\right)$	$\left(0.392\right)$	$\left(0.120\right)$	$\left(0.107\right)$	$\left(0.573\right)$	(2.7044)
$\gamma_{1}$	0.834 ***	0.852 ***	0.848 ***	0.845 ***	0.700 ***	0.713 ***	0.712 ***	0.646 ***
	$\left(0.021\right)$	$\left(0.019\right)$	$\left(0.005\right)$	$\left(0.004\right)$	$\left(0.045\right)$	$\left(0.044\right)$	$\left(0.027\right)$	$\left(0.034\right)$
$\gamma_{2}$	3.007 ***	31.229 ***	6.831 ***	5.750 ***	3.108 ***	27.584 ***	3.652 ***	1.206
	$\left(0.336\right)$	$\left(3.301\right)$	$\left(0.389\right)$	$\left(0.238\right)$	$\left(0.474\right)$	$\left(4.068\right)$	$\left(0.637\right)$	$\left(2.689\right)$
$\gamma_{2}^{*}$	1.117 **	1.453	−0.118 ***	−	1.699 ***	11.849 **	−0.184 ***	−
	$\left(0.624\right)$	$\left(8.694\right)$	$\left(0.023\right)$	−	$\left(0.435\right)$	$\left(4.799\right)$	$\left(0.027\right)$	−
$\gamma_{3}$	−	−	0.411 ***	0.564 ***	−	−	0.854 ***	3.047
	−	−	(0.018)	(0.025)	−	−	(0.133)	(2.133)

Note: The numbers in the brackets are the corresponding standard deviations. The symbol − indicates that the model excludes the corresponding parameter and the symbols *, **, and *** represent statistical significance at 10%, 5%, and 1% levels, respectively.

**Table 2:** Sequence similarity for the scale and shape parameters as estimated across the three CAcF models.

SSE50	$\left({\hat{\sigma }}_{t}^{\left(L\right)},{\hat{\sigma }}_{t}^{\left(E\right)}\right)$	$\left({\hat{\sigma }}_{t}^{\left(L\right)},{\hat{\sigma }}_{t}^{\left(S\right)}\right)$	$\left({\hat{\sigma }}_{t}^{\left(E\right)},{\hat{\sigma }}_{t}^{\left(S\right)}\right)$	$\left({\hat{\alpha }}_{t}^{\left(L\right)},{\hat{\alpha }}_{t}^{\left(E\right)}\right)$	$\left({\hat{\alpha }}_{t}^{\left(L\right)},{\hat{\alpha }}_{t}^{\left(S\right)}\right)$	$\left({\hat{\alpha }}_{t}^{\left(E\right)},{\hat{\alpha }}_{t}^{\left(S\right)}\right)$
CosD	$3.84\times 10^{-5}$	0.0001	$2.24\times 10^{-5}$	$4.99\times 10^{-5}$	0.0008	0.0010
MED	0.0021	0.0008	0.0022	0.0974	0.2791	0.2711
CSI300	$\left({\hat{\sigma }}_{t}^{\left(L\right)},{\hat{\sigma }}_{t}^{\left(E\right)}\right)$	$\left({\hat{\sigma }}_{t}^{\left(L\right)},{\hat{\sigma }}_{t}^{\left(S\right)}\right)$	$\left({\hat{\sigma }}_{t}^{\left(E\right)},{\hat{\sigma }}_{t}^{\left(S\right)}\right)$	$\left({\hat{\alpha }}_{t}^{\left(L\right)},{\hat{\alpha }}_{t}^{\left(E\right)}\right)$	$\left({\hat{\alpha }}_{t}^{\left(L\right)},{\hat{\alpha }}_{t}^{\left(S\right)}\right)$	$\left({\hat{\alpha }}_{t}^{\left(E\right)},{\hat{\alpha }}_{t}^{\left(S\right)}\right)$
CosD	$1.57\times 10^{-5}$	$4.66\times 10^{-5}$	$1.12\times 10^{-5}$	$1.73\times 10^{-5}$	0.0003	0.0003
MED	0.0034	0.0024	0.0018	0.1034	0.1487	0.1360

**Table 3:** Out-of-sample forecasting performance of the three CAcF models and the AcF model.

	SSE50				CSI300			
	CAcF-L	CAcF-S	CAcF-E	AcF	CAcF-L	CAcF-S	CAcF-E	AcF
MAE	0.0187	0.0188	0.0186	0.0195	0.0186	0.0188	0.0187	0.0195
MAPE	0.3160	0.3102	0.3143	0.3312	0.2706	0.2713	0.2709	0.2973
MCP	0.0051	0.0042	0.0052	0.0113	0.0055	0.0056	0.0068	0.1483

Note: Bold characters indicate the best prediction result.

**Table 4:** Parameter estimation results for TW50.

	Period I				Period II			
	CAcF-L	CAcF-S	CAcF-E	AcF	CAcF-L	CAcF-S	CAcF-E	AcF
μ	−0.112 ***	−0.105 ***	−0.108 ***	−2.769	−0.136 ***	−0.133 ***	−0.136 ***	−0.376
	$\left(0.007\right)$	$\left(0.006\right)$	$\left(0.007\right)$	$\left(5.428\right)$	$\left(0.009\right)$	$\left(0.005\right)$	$\left(0.010\right)$	(0.242)
$\beta_{0}$	−0.338 ***	−0.373 ***	−0.250 ***	1.957	−0.264 ***	−0.289 ***	−0.180 ***	−0.077 ***
	$\left(0.069\right)$	$\left(0.082\right)$	$\left(0.038\right)$	$\left(2.162\right)$	$\left(0.058\right)$	$\left(0.061\right)$	$\left(0.025\right)$	$\left(0.056\right)$
$\beta_{1}$	0.837 ***	0.821 ***	0.853 ***	0.872 ***	0.861 ***	0.846 ***	0.870 ***	0.897 ***
	$\left(0.033\right)$	$\left(0.039\right)$	$\left(0.024\right)$	$\left(0.112\right)$	$\left(0.029\right)$	$\left(0.032\right)$	$\left(0.020\right)$	$\left(0.022\right)$
$\beta_{2}$	0.472 ***	6.438 ***	0.065 ***	1.827	0.377 ***	4.479 ***	0.071 ***	0.020
	$\left(0.097\right)$	$\left(1.391\right)$	$\left(0.017\right)$	$\left(4.427\right)$	$\left(0.082\right)$	$\left(0.934\right)$	$\left(0.017\right)$	$\left(0.020\right)$
$\beta_{2}^{*}$	0.257 *	3.362	−0.039 ***	−	0.022	0.101	−0.011	−
	$\left(0.148\right)$	$\left(2.513\right)$	$\left(0.009\right)$	−	$\left(0.171\right)$	$\left(0.924\right)$	$\left(0.008\right)$	−
$\beta_{3}$	−	−	10.391 ***	0.012	−	−	6.738 ***	8.833
	−	−	(3.268)	(0.336)	−	−	(1.630)	(9.927)
$\gamma_{0}$	0.438 ***	0.394 ***	−5.312 ***	−3.276 *	0.454 ***	0.400 ***	−4.936 ***	−4.700 ***
	$\left(0.089\right)$	$\left(0.087\right)$	$\left(0.204\right)$	$\left(1.955\right)$	$\left(0.115\right)$	$\left(0.103\right)$	$\left(0.253\right)$	(0.184)
$\gamma_{1}$	0.850 ***	0.844 ***	0.854 ***	0.858 ***	0.844 ***	0.847 ***	0.847 ***	0.878 ***
	$\left(0.032\right)$	$\left(0.035\right)$	$\left(0.003\right)$	$\left(0.121\right)$	$\left(0.041\right)$	$\left(0.040\right)$	$\left(0.004\right)$	$\left(0.002\right)$
$\gamma_{2}$	2.939 ***	35.548 ***	5.734 ***	4.120 **	2.470 ***	25.857 ***	5.383 ***	5.183 ***
	$\left(0.585\right)$	$\left(7.388\right)$	$\left(0.212\right)$	$\left(2.034\right)$	$\left(0.601\right)$	$\left(6.032\right)$	$\left(0.263\right)$	$\left(0.176\right)$
$\gamma_{2}^{*}$	1.118	12.960	−0.079 **	−	1.090	4.186	−0.112 **	−
	$\left(0.847\right)$	$\left(10.510\right)$	$\left(0.037\right)$	−	$\left(1.248\right)$	$\left(8.918\right)$	$\left(0.051\right)$	−
$\gamma_{3}$	−	−	0.510 ***	0.747	−	−	0.462 ***	0.472 ***
	−	−	(0.062)	(0.818)	−	−	(0.066)	(0.053)

Note: The numbers in the brackets are the corresponding standard deviation. The symbol − denotes the exclusion of a parameter from the model. The symbols *, **, and *** indicate statistical significance at 10%, 5%, and 1% levels, respectively.

**Table 5:** Sequence similarity for the scale and shape parameters estimated by the three CAcF models.

Period I	$\left({\hat{\sigma }}_{t}^{\left(L\right)},{\hat{\sigma }}_{t}^{\left(E\right)}\right)$	$\left({\hat{\sigma }}_{t}^{\left(L\right)},{\hat{\sigma }}_{t}^{\left(S\right)}\right)$	$\left({\hat{\sigma }}_{t}^{\left(E\right)},{\hat{\sigma }}_{t}^{\left(S\right)}\right)$	$\left({\hat{\alpha }}_{t}^{\left(L\right)},{\hat{\alpha }}_{t}^{\left(E\right)}\right)$	$\left({\hat{\alpha }}_{t}^{\left(L\right)},{\hat{\alpha }}_{t}^{\left(S\right)}\right)$	$\left({\hat{\alpha }}_{t}^{\left(E\right)},{\hat{\alpha }}_{t}^{\left(S\right)}\right)$
CosD	$2.11\times 10^{-6}$	$1.71\times 10^{-5}$	$8.82\times 10^{-6}$	$3.41\times 10^{-6}$	0.0003	0.0003
MED	$7.97\times 10^{-5}$	0.0001	$6.17\times 10^{-5}$	0.0052	0.0102	0.0059
Period II	$\left({\hat{\sigma }}_{t}^{\left(L\right)},{\hat{\sigma }}_{t}^{\left(E\right)}\right)$	$\left({\hat{\sigma }}_{t}^{\left(L\right)},{\hat{\sigma }}_{t}^{\left(S\right)}\right)$	$\left({\hat{\sigma }}_{t}^{\left(E\right)},{\hat{\sigma }}_{t}^{\left(S\right)}\right)$	$\left({\hat{\alpha }}_{t}^{\left(L\right)},{\hat{\alpha }}_{t}^{\left(E\right)}\right)$	$\left({\hat{\alpha }}_{t}^{\left(L\right)},{\hat{\alpha }}_{t}^{\left(S\right)}\right)$	$\left({\hat{\alpha }}_{t}^{\left(E\right)},{\hat{\alpha }}_{t}^{\left(S\right)}\right)$
CosD	$1.36\times 10^{-6}$	$2.32\times 10^{-5}$	$1.35\times 10^{-5}$	$1.75\times 10^{-6}$	0.0004	0.0004
MED	$6.38\times 10^{-6}$	$5.67\times 10^{-5}$	$5.78\times 10^{-5}$	0.0004	0.0077	0.0078

**Figure A1:**
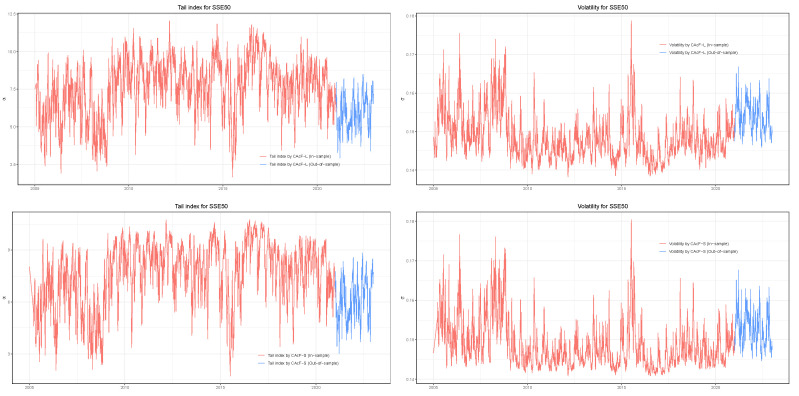
The estimated tail index $\left\{{\hat{\alpha }}_{t}\right\}$ and volatility $\left\{{\hat{\sigma }}_{t}\right\}$ by CAcF-L and CAcF-S (red line) and the corresponding predicted tail index (blue line) for the SSE50 dataset.

**Figure A2:**
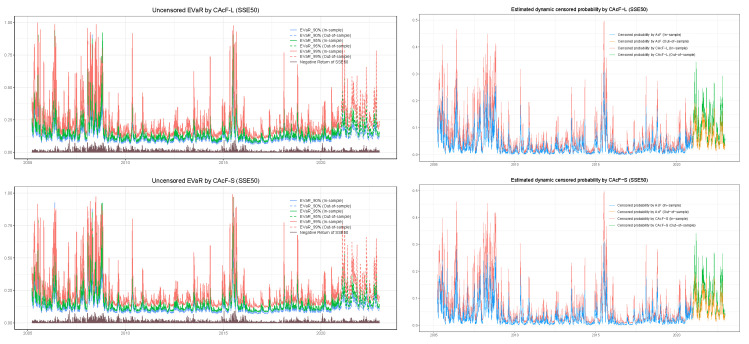
The uncensored entropic VaR and censored probability estimated via CAcF-L and CAcF-S models for the SSE50 dataset.

**Figure A3:**
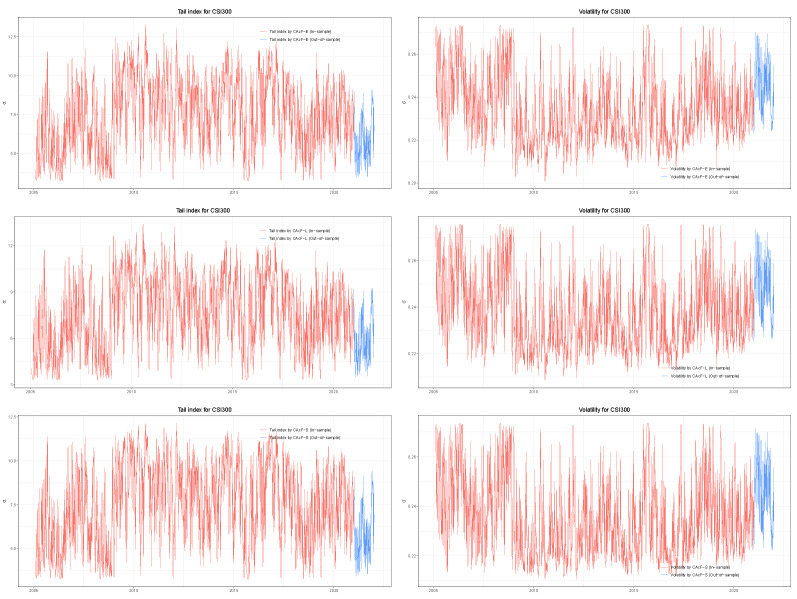
The estimated tail index $\left\{{\hat{\alpha }}_{t}\right\}$ and volatility $\left\{{\hat{\sigma }}_{t}\right\}$ by CAcF-E, CAcF-L, and CAcF-S models for the CSI300 dataset.

**Figure A4:**
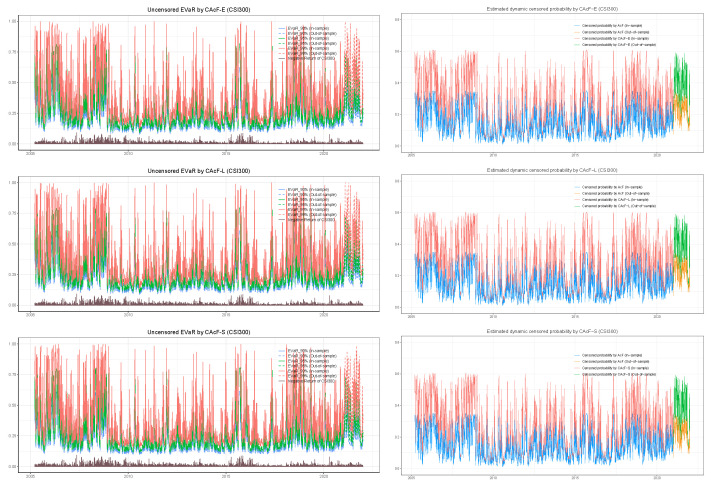
The uncensored entropic VaR and censored probability estimated via CAcF-E, CAcF-L, and CAcF-S models for the CSI300 dataset.

**Figure A5:**
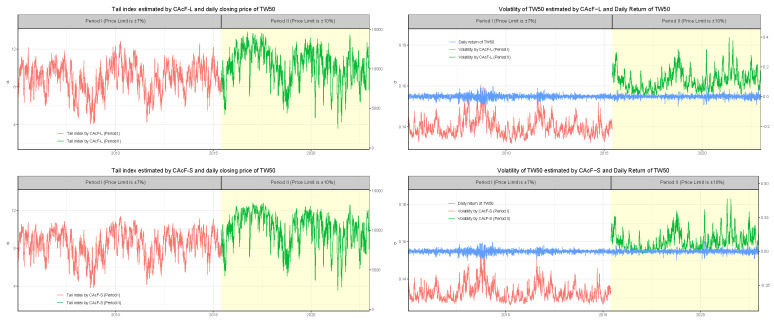
The estimated tail index $\left\{{\hat{\alpha }}_{t}\right\}$ and volatility index $\left\{{\hat{\sigma }}_{t}\right\}$ by CAcF-L and CAcF-S for the TW50 dataset. The white area represents the result of Period I and the yellow area represents the result of Period II.

**Figure A6:**
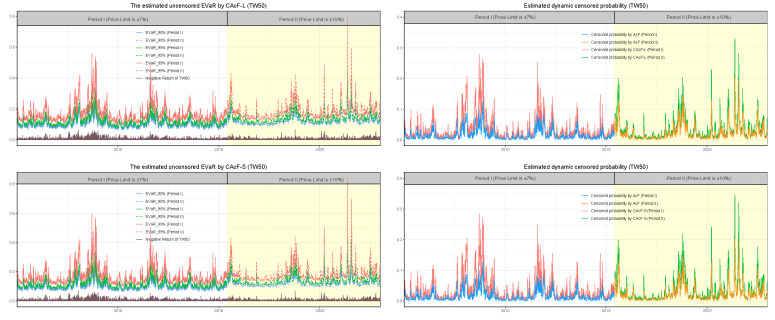
The uncensored entropic VaR and censored probability estimated via CAcF-L and CAcF-S models for the TW50 dataset. The white area represents the result of Period I and the yellow area represents the result of Period II.

## Data Availability

Data available from Yahoo Finance: https://finance.yahoo.com/ (accessed on 4 May 2024).

## Appendix A. The Significance of the Autoregression Coefficients

This section demonstrates the significance of the autoregression coefficients in model (2) concerning the μ, $\beta_{0}$, $\beta_{1}$, $\beta_{2}$, $\beta_{2}^{*}$, $\beta_{3}$, $\gamma_{0}$, $\gamma_{1}$, $\gamma_{2}$, $\gamma_{2}^{*}$, and $\gamma_{3}$ parameters of the three types of CAcF models.

**Table A1:** The significance of the autoregression coefficients in model (2).

Coefficient	Significance
μ	The location parameter in the conditional Fréchet distribution.
$\beta_{0}$	The constant term, determining the range of movement of $\left\{\log\sigma_{t}\right\}$ process.
$\beta_{1}$	The autoregression coefficient for $\left\{\log\sigma_{t}\right\}$ process.
$\beta_{2}$	The coefficient related to $G_{1}\left(Q_{t-1}\right)$, representing the impact of $Q_{t-1}$ through some observation-driven function $G_{1}\left(\cdot \right)$ on $\log\sigma_{t}$.
$\beta_{2}^{*}$	The compensation coefficient related to $G_{1}\left(Q_{t-1}\right)$ and the state $D_{t-1}$, indicating the impact of hitting limit-down on $\left\{\log\sigma_{t}\right\}$.
$\beta_{3}$	The coefficient embedded in the observation-driven function $G_{1}\left(\cdot \right)$ of exponential type for $\left\{\log\sigma_{t}\right\}$ process, which controls the shape of the function.
$\gamma_{0}$	The constant term, determining the range of movement of $\left\{\log\alpha_{t}\right\}$ process.
$\gamma_{1}$	The autoregression coefficient for $\log\alpha_{t-1}$.
$\gamma_{2}$	The coefficient related to $G_{2}\left(Q_{t-1}\right)$, representing the impact of $Q_{t-1}$ through some observation-driven function $G_{2}\left(\cdot \right)$ on $\log\alpha_{t}$.
$\gamma_{2}^{*}$	The compensation coefficient related to $G_{1}\left(Q_{t-1}\right)$ and the state $D_{t-1}$, indicating the impact of hitting limit-down on $\left\{\log\alpha_{t}\right\}$.
$\gamma_{3}$	The coefficient embedded in the observation-driven function $G_{2}\left(\cdot \right)$ of exponential type for $\left\{\log\alpha_{t}\right\}$ process, which controls the shape of the function.

## Appendix B. Proof of Proposition 1

We first present the following definition of a random variable in the Domain of Attraction of Fréchet distribution in Leadbetter et al. [35]:

**Definition A1.** 
*A random variable ε is in the Domain of Attraction of Fréchet distribution with tail index α if and only if $x_{F}=\infty$ and $1-F_{\varepsilon }\left(x\right)∼l\left(x\right)x^{-\alpha },\alpha >0$, where $F_{\varepsilon }$ is the cdf of ε,l(x) is a slowly varying function, and $x_{F}=\sup\left\{x:F_{\varepsilon }\left(x\right)<1\right\}$. Here and after, for two positive functions $m_{1}\left(x\right)$ and $m_{2}\left(x\right),m_{1}\left(x\right)∼m_{2}\left(x\right)$ means $\frac{m_{1}\left(x\right)}{m_{2}\left(x\right)}\to 1$, as x→∞.*

**Proof of Proposition 1.** In the following, the conditioning on $F_{t-1}$ is implicit and we drop the time index *t* for notation simplicity. Namely, $Q_{N}=\max_{1\leq i\leq N}X_{i}$, $a_{N}=0$, and $b_{N}={\left(\sum_{i=1}^{N}\sigma_{it}^{\alpha_{t}}\right)}^{1/\alpha_{t}}$. Denote $Q_{N}^{*}=\max_{1\leq i\leq N}X_{i}^{*}$ and set $A_{N}=\left\{\mathrm{exist}\;i\;\mathrm{such}\;\mathrm{that}\;X_{i}^{*}\geq M,1\leq i\leq N\right\}$, for any δ>0, we have

$$\begin{array}{cc} P\left(\lim_{N\to \infty }\left|Q_{N}-\max\left(Q_{N}^{*},M\right)\right|<\delta \right) & =P\left(A_{N}\right)P\left(\lim_{N\to \infty }\left|M-M\right|<\delta |A_{N}\right)\\ \; & +P\left(A_{N}^{c}\right)P\left(\lim_{N\to \infty }\left|Q_{N}^{*}-Q_{N}^{*}\right|<\delta |A_{N}^{c}\right)\\ \; & =P\left(A_{N}\right)+P\left(A_{N}^{c}\right)=1. \end{array}$$

Namely, $Q_{N}=\max\left(Q_{N}^{*},M\right),\;a.s.,\;\mathrm{as}\;N\to \infty .$ By Proposition 1 of Zhao et al. [23], we have

$$\frac{Q_{N}^{*}-a_{N}}{b_{N}}\overset{d}{⟶}\Psi_{\alpha }.$$

Then, we can conclude that

$$\frac{Q_{N}-a_{N}}{b_{N}}\overset{d}{⟶}\max\left(\Psi_{\alpha },\left(M-a_{N}\right)/b_{N}\right),$$

by the continuous mapping theorem. □

## Appendix C. The Confidence Intervals of ${\hat{\alpha }}_{t}$ and ${\hat{\sigma }}_{t}$

Here, we construct confidence intervals of ${\hat{\alpha }}_{t}$ and ${\hat{\sigma }}_{t}$ based on historical information $F_{t-2}$. Recall Equation (2); it is obvious that $Y_{t-1},Q_{t-1}\in F_{t-1}$ and $\alpha_{t-1},\sigma_{t-1}\in F_{t-2}$. Combining the monotonic relationship among $\alpha_{t}$, $\sigma_{t}$, and $Q_{t-1}$, given the historical information $F_{t-2}$, we can obtain the τ% confidence intervals for ${\hat{\alpha }}_{t}$ and ${\hat{\sigma }}_{t}$, respectively, as follows:

$$\begin{array}{cc} & \left[{\hat{\gamma }}_{0}+{\hat{\gamma }}_{1}\log{\hat{\alpha }}_{t-1}+\left({\hat{\gamma }}_{2}+{\hat{\gamma }}_{2}^{*}D_{t-1}^{(1+\tau )/2}\right){\hat{G}}_{2}\left(Q_{t-1}^{(1+\tau )/2}\right),{\hat{\gamma }}_{0}+{\hat{\gamma }}_{1}\log{\hat{\alpha }}_{t-1}+\left({\hat{\gamma }}_{2}+{\hat{\gamma }}_{2}^{*}D_{t-1}^{(1-\tau )/2}\right){\hat{G}}_{2}\left(Q_{t-1}^{(1-\tau )/2}\right)\right],\\ \mathrm{and}\; & \left[{\hat{\beta }}_{0}+{\hat{\beta }}_{1}\log{\hat{\sigma }}_{t-1}+\left({\hat{\beta }}_{2}+{\hat{\beta }}_{2}^{*}D_{t-1}^{(1-\tau )/2}\right){\hat{G}}_{1}\left(Q_{t-1}^{(1-\tau )/2}\right),{\hat{\beta }}_{0}+{\hat{\beta }}_{1}\log{\hat{\sigma }}_{t-1}+\left({\hat{\beta }}_{2}+{\hat{\beta }}_{2}^{*}D_{t-1}^{(1+\tau )/2}\right){\hat{G}}_{1}\left(Q_{t-1}^{(1+\tau )/2}\right)\right], \end{array}$$

where $D_{t}^{\tau }=I\left[Q_{t}^{\tau ,*}>M\right]$, $Q_{t}^{\tau }=\min\left(M,Q_{t}^{\tau ,*}\right)$, and $Q_{t}^{\tau ,*}=\hat{\mu }+{\hat{\alpha }}_{t}{\left(-\log\tau \right)}^{-1/{\hat{\sigma }}_{t}}$ is τ-quantile of $Q_{t}^{*}$.

## Appendix D. Appendix of Empirical Results

### Appendix D.1. Complementary Empirical Results for SSE50 and CSI300

We present here complementary empirical results for the SSE50 and CSI300 datasets. The two graphs on the left side in Figure A1 show the in-sample fitting and out-of-sample forecasts of the tail index $\left\{{\hat{\alpha }}_{t}\right\}$ in the SSE50 based on the CAcF-L and CAcF-S models. Correspondingly, the two graphs on the right show the in-sample fitting and out-of-sample predicted volatility $\left\{{\hat{\sigma }}_{t}\right\}$. These results are highly similar to Figure 2 and Figure 3, and further verify that the different CAcF models have a consensus on the estimated $\left\{{\hat{\alpha }}_{t}\right\}$ and $\left\{{\hat{\sigma }}_{t}\right\}$ processes, but different CAcF models decompose the two processes from different risk preference perspectives. Thus, although investors have different risk preferences, the risk factors embedded in the market are clear, only with different interpretations. The two graphs on the left in Figure A2 show the in-sample fitting and out-of-sample predicted results of the CAcF-L and CAcF-S models for the entropic value at risk of $Q_{t}^{*}$ in SSE50. The brown line indicates the negative daily returns of the SSE50 index (the positive daily returns of the SSE50 index are cut off to reflect the downward risk better). Correspondingly, the two graphs on the right show the in-sample fitting and out-of-sample predicted censored probability. Similarly, this is consistent with the results presented in Figure 4 and Figure 5. The straight fact is that, if the censored features of the data are not accounted for, the tail risks in the price-limited market will be underestimated.

In addition, we also present the results for the CSI300 dataset, as shown in Figure A3 and Figure A4. These results are consistent with those of the SSE50 dataset, which demonstrates that our conclusions are also applicable to the CSI300 dataset.

### Appendix D.2. Complementary Empirical Results for TW50

This section presents complementary empirical results for the TW50 index based on the CAcF-L and CAcF-S models. As shown in Figure A5 and Figure A6, these results are highly similar to the results based on the CAcF-E model (see more details in Section 3.2).
